# Osteoporotic fractures prediction in Chinese postmenopausal women: a machine learning-based multi-dimensional approach

**DOI:** 10.3389/fendo.2026.1788788

**Published:** 2026-04-17

**Authors:** Wei Zhu, Yang Guo, Jiang Shuai, Longwang Tan, Chuang Liu, Yongjun Jia, Chi Zhang, Kok-Yong Chin

**Affiliations:** 1 Department of Orthopedics, Affiliated Hospital of Xizang Mizu University, Xianyang, Shaanxi, China; 2 Department of Nursing, School of Medicine, Shaanxi Institute of International Trade & Commerce, Xianyang, Shaanxi, China; 3 Medical Morphology Experiment Centre, School of Basic Medicine, Hunan University of Medicine, Huaihua, Hunan, China; 4 Division of Spinal Surgery, Department of Nursing, Affiliated Hospital of Shaanxi University of Chinese Medicine, Xianyang, Shaanxi, China; 5 Rehabilitation Department (Area 8), Xi’an Daxing Hospital, Xi’an, Shaanxi, China; 6 Department of Pharmacology, Faculty of Medicine, University Kebangsaan Malaysia, Cheras, Malaysia

**Keywords:** bone mineral density, bone turnover markers, machine learning, osteoporotic fracture, postmenopausal women, risk prediction

## Abstract

Osteoporotic fractures are a major complication of osteoporosis and pose a substantial global health burden, particularly in postmenopausal women. Although bone mineral density (BMD) is widely used for fracture risk assessment, its predictive accuracy is limited, and integrating multidimensional clinical indicators may improve risk prediction. This retrospective study included 1,717 postmenopausal women from two tertiary hospitals in Shaanxi Province, China, who were classified into fracture (n=797) and non-fracture (n=920) groups based on a history of low-energy fractures. Thirty-two clinical variables, including BMD, bone turnover markers (BTMs), serum electrolytes, age, and body mass index, were analyzed. Recursive feature elimination was applied, and ten machine learning models were developed using a training dataset (70%) and evaluated on a testing dataset (30%). Model performance was assessed using the area under the receiver operating characteristic curve (AUC), accuracy, sensitivity, and specificity. Model interpretability was explored using SHapley Additive exPlanations (SHAP). Among all models, the Random Forest model demonstrated the best performance (AUC = 0.872), outperforming the Extra Trees (AUC = 0.841) and XGBoost (AUC = 0.836) models. SHAP analysis identified BMD, serum chloride (Cl^-^), age, albumin-to-globulin ratio, and neutrophil percentage as the most influential predictors, with osteocalcin N-mid fragment contributing more prominently than other BTMs. In conclusion, this machine learning-based model effectively identified key risk factors for osteoporotic fractures in postmenopausal women, and integrating BMD with biochemical and clinical indicators may improve fracture risk prediction and support clinical screening and risk stratification.

## 1 Introduction

Osteoporosis (OP) is a systemic metabolic bone disease characterized by reduced bone mass and deterioration of bone microarchitecture, leading to increased bone fragility and an elevated risk of fractures with advancing age (1). In China, 49 million women and 22.8 million men over the age of 50 years suffer from osteoporosis (2). Osteoporotic fractures, as the most severe complication of osteoporosis, pose a major global public health challenge due to their high disability and mortality rates. In 2010, the total number of individuals with osteoporotic fractures worldwide reached 158 million, with 8.9 million new cases occurring annually. It is projected that by 2040, this number will increase to 300 million globally (3). Furthermore, the economic burden attributed to osteoporotic fractures accounts for approximately 0.83% of the global costs of non-communicable diseases (4). Among women over the age of 55, the inpatient burden and hospitalization costs associated with osteoporotic fractures significantly exceed those of stroke, myocardial infarction, and breast cancer (5). The annual economic burden of osteoporotic fractures in the United States has increased significantly, from USD 19 billion in 2013 to USD 25.3 billion in 2023 (6). By 2040, the fracture-related medical costs will reach USD 50 billion (7, 8). In China, the annual number of osteoporotic fractures and related treatment costs will double by 2035. By 2050, the total number of fractures is projected to reach 5.99 million, with costs expected to exceed USD 25.43 billion (9).

Bone mineral density (BMD) measured by dual-energy X-ray absorptiometry (DXA) is the most widely used clinical tool for fracture risk assessment (10, 11). However, its predictive accuracy is inherently limited. First, BMD reflects only the quantity of mineralized bone, but cannot capture bone quality, including microarchitecture, material properties, and turnover dynamics (12). Second, artefacts such as spinal degenerative osteophytes, aortic calcification, or vertebral compression fractures often spuriously elevate BMD readings in older adults, leading to underestimation of fracture risk (13, 14). Finally, a substantial proportion of fragility fractures occur in individuals with BMD T-scores above the osteoporosis threshold (T-scores > –2.5), indicating that BMD alone misses clinically significant bone fragility (15, 16). Although complementary tools such as trabecular bone score (TBS) can partially assess bone microarchitecture (17), they are not yet universally integrated into routine practice. Thus, sole reliance on BMD contributes to the underdiagnosis of high-risk individuals (18).

In recent years, bone turnover markers (BTMs) have attracted attention as potential complementary tools for fracture risk assessment. BTMs are non-invasive biomarkers that reflect the dynamic state of bone metabolism and are generally divided into formation markers (e.g., procollagen type I N-terminal propeptide [P1NP], osteocalcin) and resorption markers (e.g., C-terminal telopeptide of type I collagen [CTX-1]) (19). Elevated bone resorption rates, indicated by increased CTX-1 or undercarboxylated osteocalcin, have been independently associated with two- to three-fold higher risk of hip and vertebral fractures in postmenopausal women, even after adjusting for BMD and age (20, 21). The International Osteoporosis Foundation (IOF) and International Federation of Clinical Chemistry and Laboratory Medicine (IFCC) recommend serum CTX-1 and P1NP as reference markers for fracture risk prediction and treatment monitoring (22). However, clinical adoption of BTMs has been limited by pre-analytical variability (circadian rhythms, food intake) and the lack of standardized reference ranges (23, 24). Therefore, identifying which BTMs most robustly add predictive value beyond BMD remains an active research priority.

With the rapid advancement of artificial intelligence (AI) in clinical research, machine learning (ML) techniques are increasingly used to extract complex patterns from large-scale electronic medical records (EMRs) and to support clinical decision-making (20, 21). ML-based prediction models have demonstrated favorable performance in various chronic diseases (22-25), yet the development and validation of such models for osteoporotic fracture risk in postmenopausal women remain an area of active investigation. Most existing models rely heavily on BMD and basic clinical variables, and the added value of incorporating multiple BTMs, electrolytes, and inflammatory markers has not been systematically evaluated.

Therefore, the primary objective of this study was to develop and validate a ML-based prediction model that integrates multidimensional clinical features, including lumbar spine BMD, BTMs (P1NP, β-CTX, and osteocalcin N-mid fragment [N-MID]), serum electrolytes, and complete blood count parameters to estimate osteoporotic fracture risk in postmenopausal women. The secondary objective was to identify the most influential predictors and to evaluate the relative contribution of N-MID compared with IOF/IFCC-recommended BTMs using SHapley Additive exPlanations (SHAP). We hypothesized that a comprehensive model combining bone density, biochemical, and systemic indicators would outperform models based on BMD alone, and that N-MID would emerge as a non-inferior or superior predictor relative to β-CTX and P1NP.

## 2 Materials and methods

### 2.1 Study design and subjects

This was a retrospective study designed to develop and validate the association between various BTMs and BMD in postmenopausal female patients, as well as to establish a predictive model for osteoporotic fracture risk. The study included postmenopausal female patients from the orthopedic departments of two tertiary hospitals in Shaanxi Province, China, between January 2022 and June 2025. A total of 1,717 subjects were selected based on inclusion and exclusion criteria. The study was approved by the Ethics Committee of the Medical College, Shaanxi Institute of International Commerce and Trade (Approval No.: YYXY-HLX-2024-12-28).

This retrospective study identified eligible patients with osteoporotic fractures from the electronic medical record (EMR) system of two hospitals. The diagnosis of osteoporotic fractures this study follows the definition by World Health Organization (WHO) (1994) (26), which defined as a fracture that occurs due to low-energy trauma, such as a fall from standing height or less, that would not normally cause a fracture in healthy bone. It reflects reduced bone strength, typically due to osteoporosis, and commonly involves the hip, vertebrae, distal radius, or proximal humerus. The osteoporotic fracture case was identified from radiology reports and orthopedic consultation records, wherein the fracture sites and types were verified. Based on clinical notes, high-energy trauma cases (e.g., motor vehicle accidents, falls from height >1 m, or sports injuries) were excluded, retaining only low-energy fall-related fractures.

The diagnosis of osteoporosis of this study follows the definition by World Health Organization (WHO) (1994), which determine osteoporosis based on BMD measured by DXA at lumbar spine (L1–L4). A T-score ≤ -2.5 standard deviations (SD) below the peak bone mass of healthy women aged 20–30 years was classified as osteoporosis. A T-score between -1.0 and -2.5 SD was defined as osteopenia or low bone mass.

Participants eligible for inclusion were postmenopausal women aged 45 years or older with a confirmed diagnosis of primary osteoporosis. A prerequisite for enrolment was the availability of complete clinical data, including DXA scans, laboratory results, and BMI measurements. All subjects included were treatment-naïve for osteoporosis. The fracture group was defined as participants who had sustained a recent fracture resulting from low-energy trauma.

Subjects were excluded if they were presented with any conditions known to cause secondary osteoporosis. These included metabolic bone diseases (e.g., osteomalacia, Paget’s disease), endocrine disorders (e.g., hyperparathyroidism, Cushing’s syndrome, hyperthyroidism), hematological diseases, and connective tissue disorders (e.g., rheumatoid arthritis, lupus erythematosus). Further exclusion criteria were the presence of bone tumors, chronic renal failure, or the current use of medications affecting bone metabolism, such as hormone therapy, glucocorticoids, thyroid supplements, anticonvulsants, or warfarin. Patients with metallic implants that could interfere with DXA imaging, those with incomplete clinical data, or those whose fractures were pathological (i.e., due to tumors) or resulted from high-energy trauma were also excluded from the study.

The electronic medical record (EMR) systems of the two participating hospitals were reviewed to collect comprehensive patient information, including a history of osteoporotic fractures within the past decade, demographic characteristics (age, height, weight, BMI), medical history, medication use, and surgical history. Laboratory data were retrieved from the hospital databases, encompassing bone turnover markers (MAGLUMI-X6, Shenzhen New Industry Biomedical Engineering Co., Ltd., Shenzhen, China), serum vitamin D [25(OH)D] levels (Abbott A3600, Abbott Diagnostics, Illinois, USA), complete blood count (Mindray BC-5309, Shenzhen Mindray Bio-Medical Electronics Co., Ltd., Shenzhen, China), as well as serum protein and electrolyte parameters (Hitachi 7600, Hitachi High-Tech, Tokyo, Japan).

BMD data were extracted from DXA examination reports archived in the EMRs, all measured at the lumbar spine (L1-L4). Both hospitals utilized the same DXA device for BMD assessment (MEDIX-DR, Medilink/DMS Imaging, Le Montueux, France). When BMD records were unavailable in the EMR, the imaging departments were contacted to identify the corresponding DXA results using the patient’s full name or admission number.

### 2.2 Feature selection

To identify the optimal subset of predictors from the 30 initial clinical variables, we applied recursive feature elimination (RFE) with 10-fold cross-validation on the training set (n=1,201). RFE was performed using ten classifiers representing different algorithmic principles: Logistic Regression (LR), Support Vector Machine with RBF kernel (SVM), Decision Tree (DT), Random Forest (RF), Extra Trees (ET), Gradient Boosting (GB), AdaBoost (ADA), XGBoost (XGB), K-Nearest Neighbors (KNN), and Naive Bayes (NB). For each classifier, RFE iteratively removed the least important feature and evaluated model performance (AUC) across different feature subset sizes. The cross-validation results indicated that for all ten classifiers, performance plateaued or marginally increased when all 30 features (excluding two redundant identifiers) were retained; no smaller subset achieved consistently higher AUC. Therefore, all 30 clinical predictors were included in the final model development. The training and testing sets were randomly split in a 7:3 ratio (27, 28), and ten-fold cross-validation was integrated into the training process to enhance generalizability and robustness (29, 30).

### 2.3 Model validation and feature importance interpretation

Model performance was comprehensively evaluated using standard metrics, including AUC, accuracy, sensitivity, and specificity. Clinical utility was further assessed through calibration curves and decision curve analysis, examining the alignment between predicted probabilities and observed outcomes across various risk thresholds. We applied SHAP to quantify the contribution of each variable to the model’s predictions (20). This approach enabled transparent interpretation of feature importance and their directional effects on osteoporosis diagnosis.

### 2.4 Statistical analysis

Statistical analyses were performed using SPSS 26.0 (IBM, Armonk, NY, USA) and Python 3.10.1 (Python Software Foundation, 2021). Continuous and categorical variables were compared using appropriate parametric and non−parametric tests. Multicollinearity among predictors was assessed using variance inflation factors. All tests were two−tailed with statistical significance set at P<0.05.

## 3 Results

### 3.1 Baseline data evaluation

A total of 1,717 patients were included based on the eligibility criteria. Among them, 797 patients (46.42%) had experienced fractures within the past 10 years, while 920 patients (53.58%) had no history of fractures. All clinical data were obtained from electronic medical records, totaling 32 clinical features. Detailed characteristics are presented in **Table 1**. The cohort was randomly stratified into a training set (n = 1,201) and a test set (n = 516) at a ratio of 7:3. No statistically significant differences were observed in the distribution of variables between these two subsets.

**Table 1:** Baseline characteristics of patients with osteoporotic fracture.

Variable	Overall (1717)	No-fracture (920)	Fracture (797)	Statistics	P-value
Age (year)	67.86 ± 8.44	65.67 ± 8.15	70.39 ± 8.07	247169.5	<0.001
BMI (kg/cm^2^)	23.62 ± 3.40	23.93 ± 3.27	23.25 ± 3.50	410095	<0.001
BMD	0.75 ± 0.15	0.80 ± 0.15	0.70 ± 0.14	503727	<0.001
25-(OH)D (ng/mL)	36.80 ± 19.28	35.15 ± 19.51	38.71 ± 18.85	320059.5	<0.001
N-MID (ng/mL)	17.86 ± 13.75	17.37 ± 15.56	18.42 ± 11.30	309538.5	<0.001
P1NP (ng/mL)	62.85 ± 29.39	61.49 ± 26.65	64.43 ± 32.20	352999.5	0.184
β-CTX (ng/mL)	1.12 ± 19.08	0.64 ± 0.35	1.66 ± 28.00	345664.5	0.041
WBC (10^9^/L)	5.54 ± 1.97	5.57 ± 1.97	5.51 ± 1.97	372223	0.585
NEUT (%)	60.28 ± 11.50	58.05 ± 11.33	62.86 ± 11.15	270432	<0.001
RBC (10^12^/L)	4.24 ± 2.86	4.36 ± 3.60	4.10 ± 1.61	446264.5	<0.001
HBG (g/L)	123.92 ± 15.80	125.32 ± 15.62	122.31 ± 15.86	425793.5	<0.001
HCT (%)	38.53 ± 5.38	38.96 ± 4.85	38.02 ± 5.90	420361	<0.001
PTH (g/L)	199.53 ± 65.85	209.13 ± 62.54	188.45 ± 67.84	436652.5	<0.001
TP (g/L)	66.75 ± 15.31	67.16 ± 19.93	66.29 ± 6.81	378006.5	0.266
ALB (g/L)	39.48 ± 4.38	40.46 ± 4.19	38.36 ± 4.32	472356.5	<0.001
GLB (g/L)	26.88 ± 4.87	26.02 ± 4.50	27.87 ± 5.09	282605	<0.001
A/G	1.52 ± 0.33	1.60 ± 0.33	1.42 ± 0.31	495417.5	<0.001
FPG (mmol/L)	5.44 ± 1.51	5.57 ± 1.62	5.29 ± 1.35	415545	<0.001
Na^+^ (mmol/L)	142.39 ± 3.89	142.87 ± 4.50	141.82 ± 2.96	459113.5	<0.001
CL^-^ (mmol/L)	106.21 ± 4.51	105.60 ± 5.02	106.92 ± 3.71	260531	<0.001
Ca^2+^ (mmol/L)	2.27 ± 0.15	2.29 ± 0.14	2.25 ± 0.16	435781.5	<0.001
Menopausal period					
≤10 year	374(21.8%)	271(29.5%)	103(12.9%)	68.513	<0.001
>10 year	1343(78.2%)	649(70.5%)	694(87.1%)		
Smoking					
Yes	3(0.2%)	3(0.3%)	0(0.0%)	12098.1	0.253
No	1714(99.8%)	917(99.7%)	797(100.0%)		
Drinking					
Yes	0(0.0%)	0(0.0%)	0(0.0%)	–	1.000
No	1717(100.0%)	920(100.0%)	797(100.0%)		
Surgical history					
Yes	1057(61.6%)	494(53.7%)	563(70.6%)	51.813	<0.001
No	660(38.4%)	426(46.3%)	234(29.4%)		
Antihypertensive drug use					
Yes	1695(98.7%)	914(99.3%)	781(98.0%)	6.202	0.013
No	22(1.3%)	6(0.7%)	16(2%)		
Hypoglycemic drug use					
Yes	489(28.5%)	296(32.2%)	193(24.2%)	13.278	<0.001
No	1228(71.5%)	624(67.8%)	604(75.8%)		
Lipid-lowering drug use					
Yes	144(8.4%)	93(10.1%)	51(6.4%)	7.649	0.006
No	1573(91.6%)	827(89.9%)	746(93.6%)		
Other drug use					
Yes	187(10.9%)	130(14.1%)	57(7.2%)	21.43	<0.001
No	1530(89.1%)	790(85.9%)	740(92.8%)		
Presence of other diseases					
Yes	291(16.9%)	142(15.4%)	149(18.7%)	3.225	0.073
No	1426(83.1%)	778(84.6%)	648(81.3%)		

BMD, bone mineral density; 25-(OH)D, 25-hydroxyvitamin D; N-MID, N-terminal mid-fragment of osteocalcin; P1NP, procollagen type I N-terminal propeptide; BALP, bone-specific alkaline phosphatase; β-CTX, β-cross-linked C-telopeptide of type I collagen; WBC, white blood cell count; NEUT%, neutrophil percentage; RBC, red blood cell count; HBG, hemoglobin; HCT, hematocrit; PC, platelet count; TP, total protein; ALB, albumin; GLB, globulin; A/G, albumin/globulin ratio; GLU, glucose; K^+^, potassium ion; Na^+^, sodium ion; CL, chloride ion; Ca^2+^, calcium ion.

### 3.2 Feature selection

**Figure 1:**
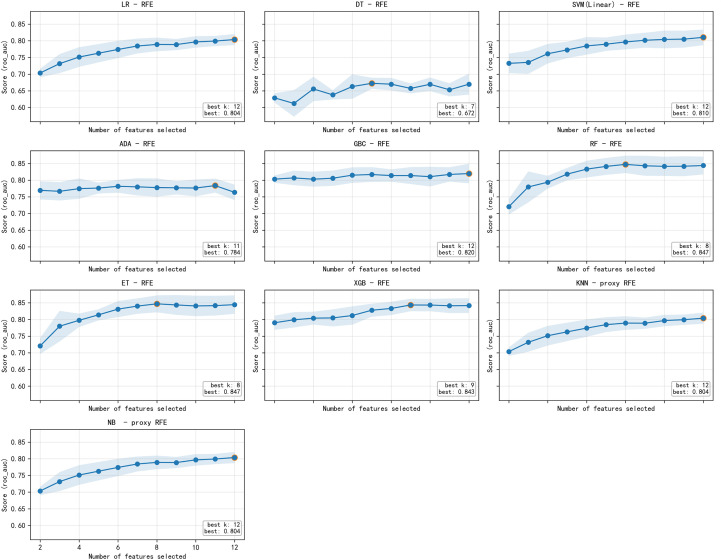
Results of nine different algorithms using recursive feature elimination (RFE) procedure for feature selection. LR, logistic regression, class_weight=balanced; SVM, support vector machine with RBF kernel; DT, decision tree; RF, random forest; ET, extra trees; GB, gradient boosting; ADA, AdaBoost; XGB, XGBoost; KNN, k-nearest neighbors; NB, naive Bayes.

To identify the optimal subset of predictors, RFE with 10-fold cross-validation was applied to the training set using ten classifiers (LR, SVM, DT, RF, ET, GB, ADA, XGB, KNN, NB). **Figure 1** illustrates the cross-validated AUC as a function of the number of features for each algorithm. For all classifiers, performance plateaued when the full set of 30 clinical predictors was retained; no smaller subset yielded consistently higher AUC. Therefore, all 30 features were included in the final model development.

### 3.3 Model performance for fracture prediction

**Figure 2:**
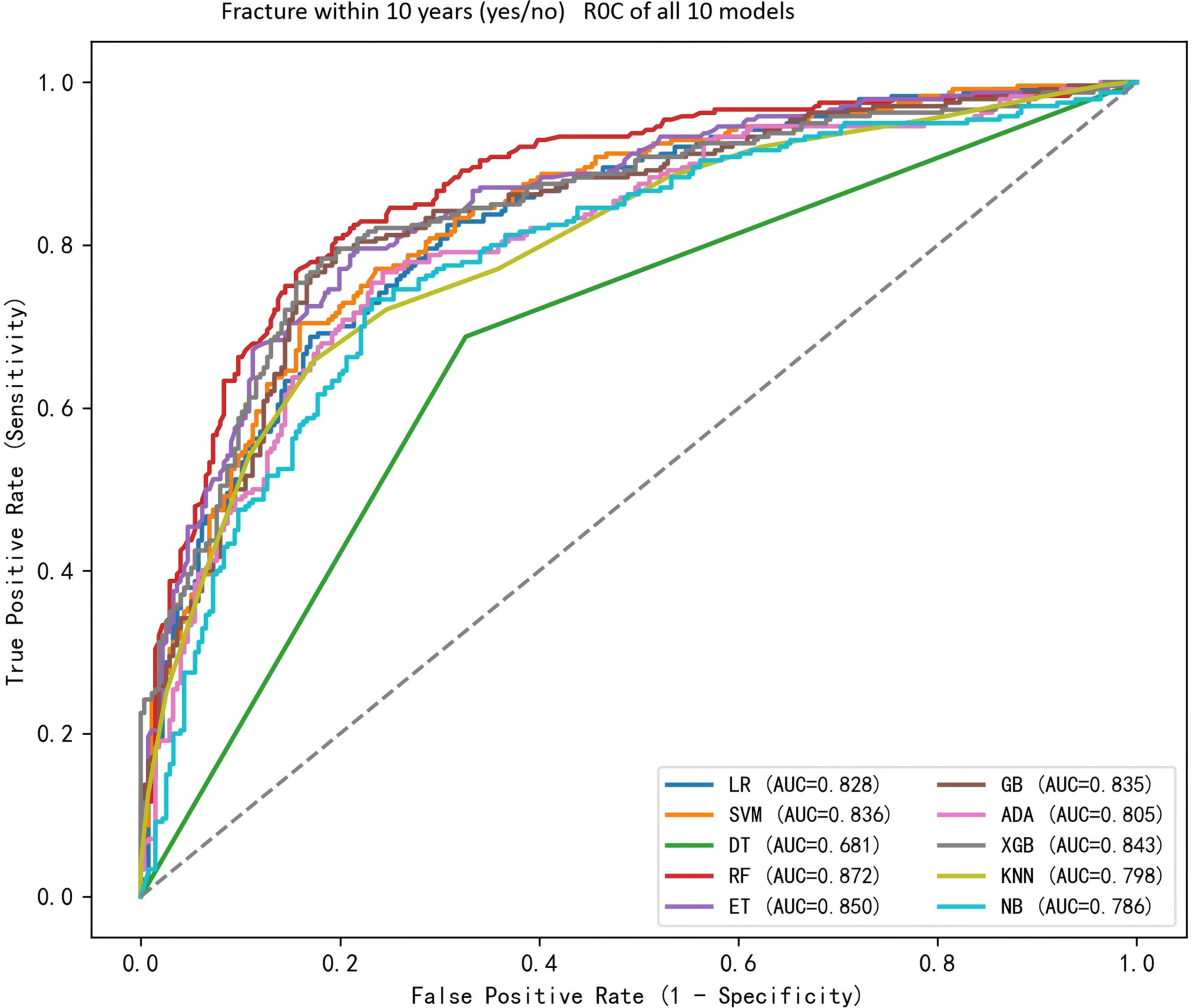
Comparison of the area under the receiver operating characteristic curves for 10 machine learning algorithms. LR, logistic regression, class_weight=balanced; SVM, support vector machine with RBF kernel; DT, decision tree; RF, random forest; ET, extra trees; GB, gradient boosting; ADA, AdaBoost; XGB, XGBoost; KNN, k-nearest neighbors; NB, naive Bayes.

The performance of the ten machine learning models on the independent test set is summarized in **Table 2**. Among all algorithms, RF achieved the highest discriminative ability with an AUC of 0.872, followed by ET (AUC = 0.841) and XGB (AUC = 0.836). The corresponding accuracy, sensitivity, and specificity are detailed in **Table 2**. **Figure 2** presents the receiver operating characteristic (ROC) curves for all ten models, visually confirming the superior performance of RF. Based on these results, RF, ET, and XGB were selected as base models for subsequent ensemble modeling and in-depth analysis.

**Table 2:** Comparison of the prediction results of each test model using test datasets.

Model	CV_AUC	AUC	ACC	Sens	Spec	PPV	NPV	F1
RF	0.834	0.872	0.795	0.779	0.808	0.779	0.808	0.779
ET	0.817	0.841	0.767	0.738	0.793	0.756	0.777	0.747
XGB	0.822	0.836	0.785	0.779	0.79	0.763	0.804	0.771
SVM	0.816	0.83	0.775	0.783	0.768	0.746	0.803	0.764
GB	0.799	0.826	0.766	0.767	0.764	0.739	0.79	0.753
LR	0.784	0.814	0.748	0.750	0.746	0.720	0.774	0.735
ADA	0.752	0.802	0.752	0.762	0.743	0.720	0.782	0.741
KNN	0.726	0.773	0.481	0.979	0.047	0.472	0.722	0.637
NB	0.749	0.772	0.700	0.75	0.656	0.655	0.751	0.699
DT	0.653	0.652	0.653	0.638	0.667	0.624	0.679	0.631

LR, logistic regression, class_weight=balanced; SVM, support vector machine with RBF kernel; DT, decision tree; RF, random forest; ET, extra trees; GB, gradient boosting; ADA, AdaBoost; XGB, XGBoost; KNN, k-nearest neighbors; NB, naive Bayes.

### 3.4 Model optimization and comprehensive evaluation

(1) Hyperparameter tuning and cross-validation.

**Figure 3:**
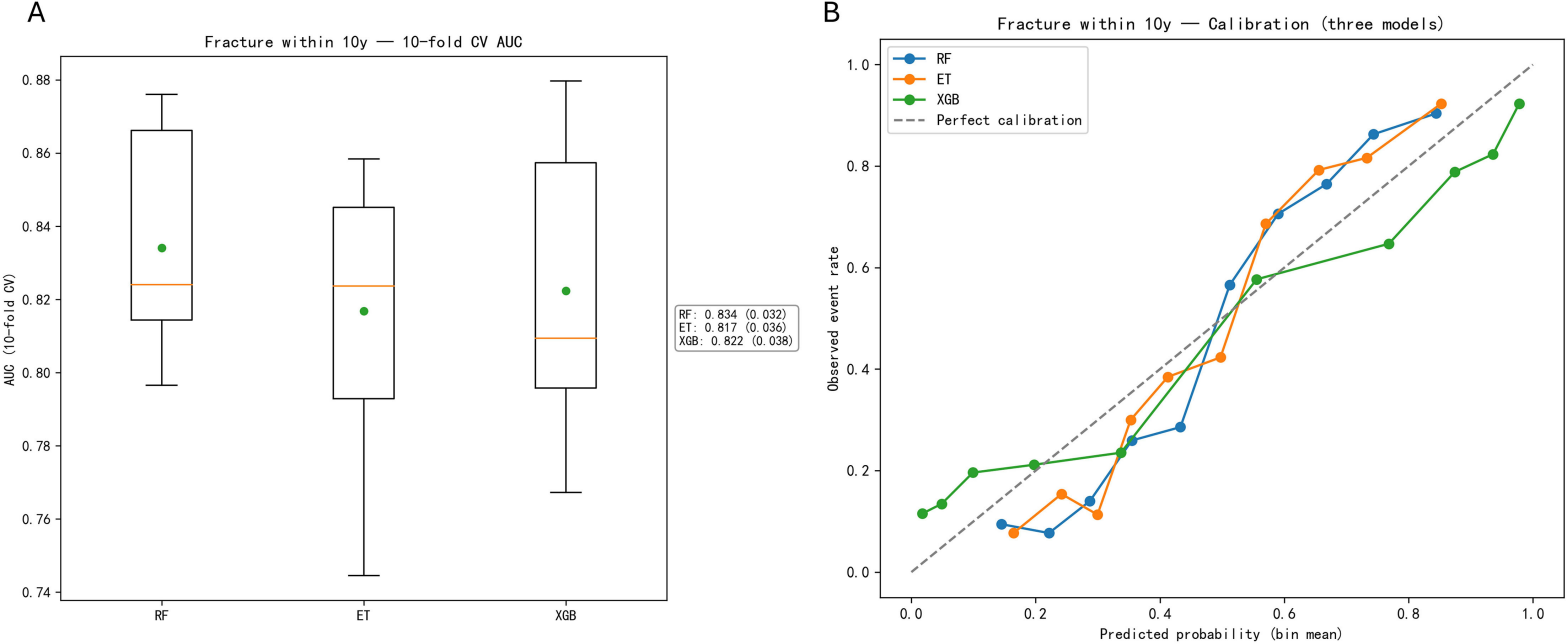
Comparative performance and calibration of machine learning models for predicting fracture risk. The figure illustrates the predictive capabilities of Random Forest (RF), Extra Trees (ET), and Extreme Gradient Boosting (XGB) through two primary analyses: **(A)** a boxplot of 10-fold cross-validation Area Under the Curve (AUC) scores, where RF exhibits the highest mean performance (AUC = 0.834) followed by XGB and ET; and **(B)** a calibration curve comparing predicted probabilities against observed event rates, showing that while all models generally follow the line of perfect calibration, RF and ET demonstrate closer alignment to observed outcomes in higher-risk bins compared to the relative under-prediction seen in the XGB model.

The three selected algorithms (RF, ET, XGB) underwent hyperparameter optimization via randomized search with 10-fold cross-validation on the training set. The internal validation AUC scores are presented as boxplots in **Figure 3A**, demonstrating that the optimized RF classifier maintained superior predictive capability (mean CV-AUC = 0.834) compared to ET (0.817) and XGB (0.822) (**Figure 3B**).

(2) Calibration and clinical utility.

**Figure 4:**
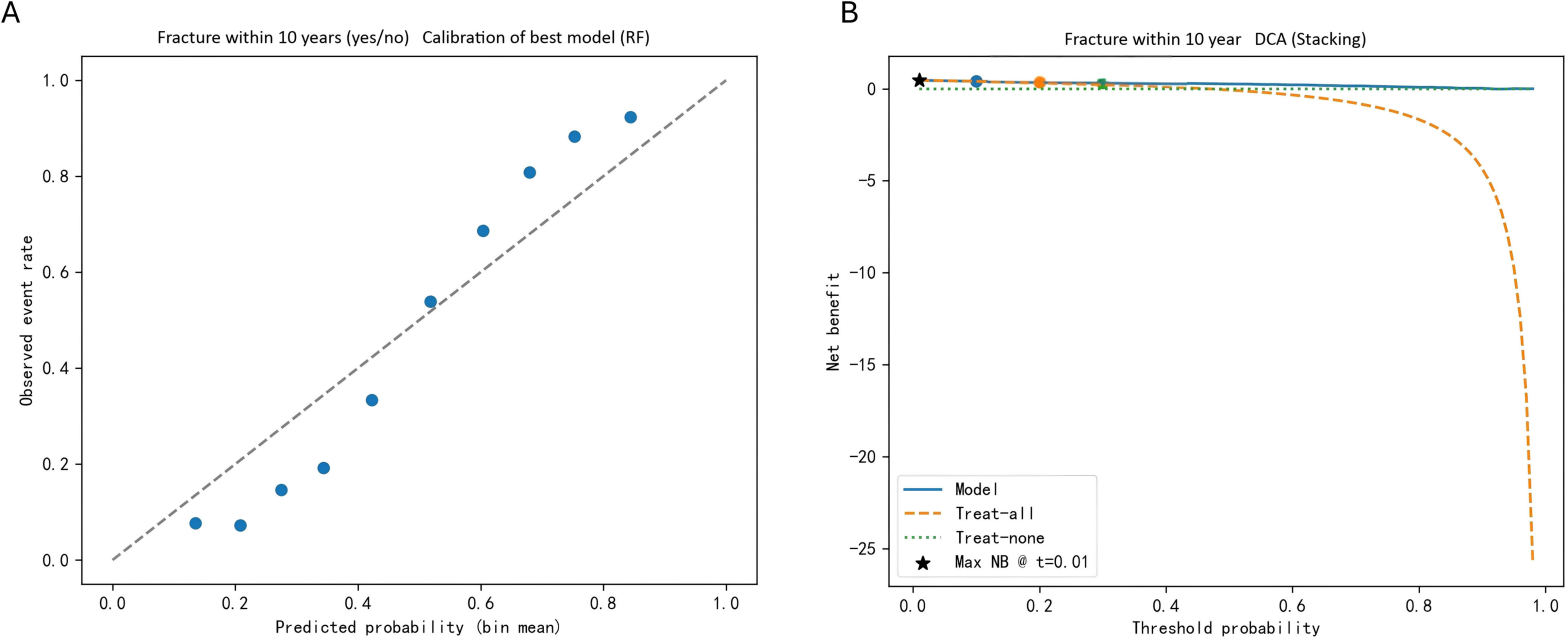
Model calibration and clinical utility for fracture prediction: **(A)** calibration plot of the best-performing random forest (RF) model demonstrating good agreement between predicted probabilities and observed fracture rates across risk strata. Minor deviations are observed at lower predicted risk levels. **(B)** Decision curve analysis (DCA) of the stacking model reveals a higher net benefit than both the treat-all and treat-none strategies across a wide range of clinically relevant threshold probabilities, with the maximum net benefit occurring at a low threshold (≈0.01), supporting the potential clinical usefulness of the model for fracture risk stratification.

Calibration curves were constructed to assess the agreement between predicted fracture probabilities and observed outcomes. The RF model exhibited excellent calibration, with predictions closely aligning with the ideal diagonal line (**Figure 4A**). Decision curve analysis (DCA) confirmed the clinical usefulness of the stacking model, showing a higher net benefit than both the treat−all and treat−none strategies across a wide range of clinically relevant threshold probabilities (**Figure 4B**).

(3) Test set performance of the final RF model.

**Figure 5:**
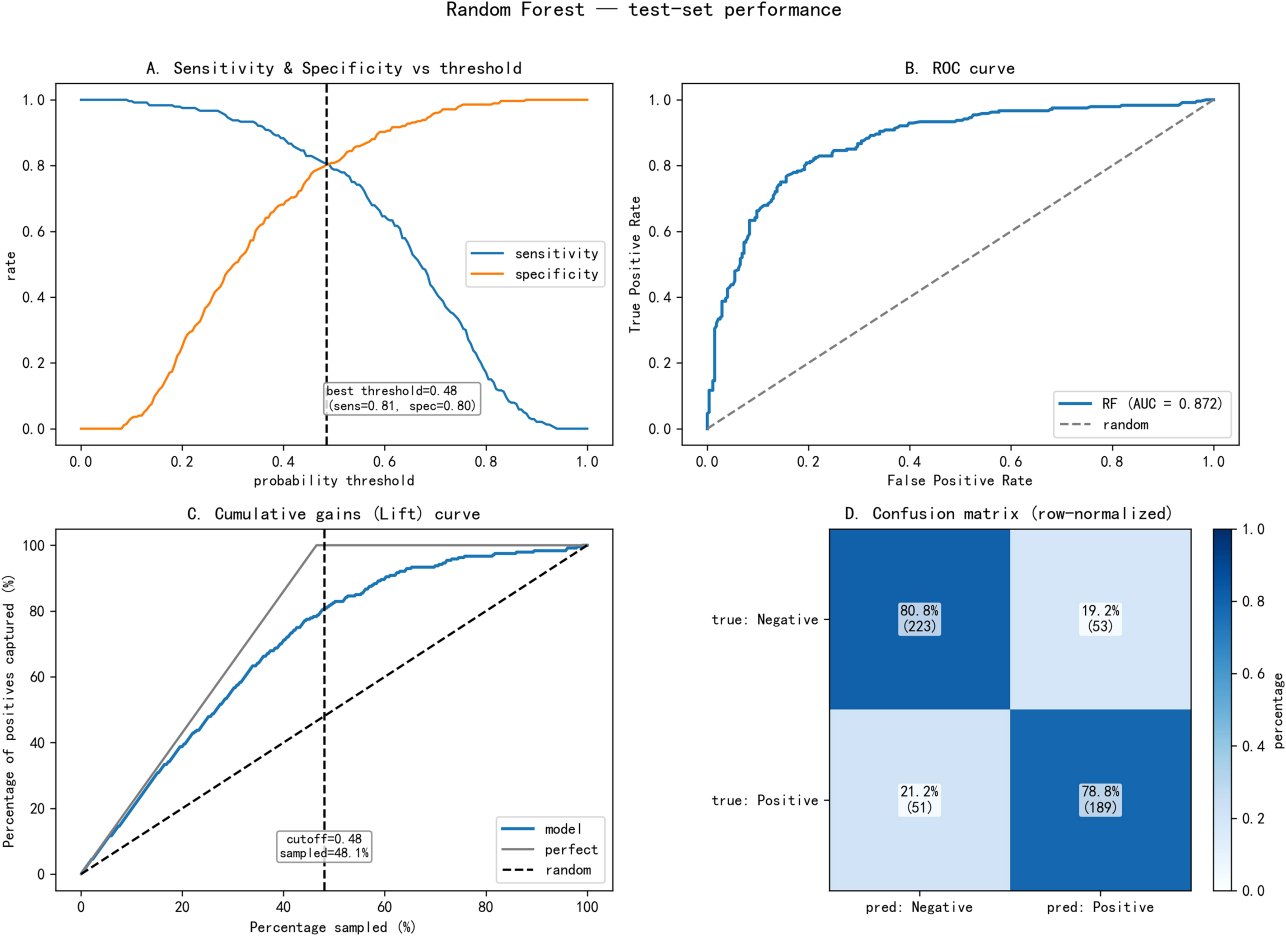
Random Forest test-set performance. The classification performance was evaluated through four key analytical lenses: **(A)** the relationship between probability thresholds and model sensitivity/specificity, identifying an optimal cutoff at 0.48; **(B)** the Receiver Operating Characteristic (ROC) curve, which demonstrates a high discriminative power with an AUC of 0.872; **(C)** a Cumulative Gains (Lift) curve showing that the model captures approximately 80% of total positives within the first 48.1% of the sampled population; and **(D)** a row-normalized confusion matrix illustrating a balanced classification accuracy, specifically correctly identifying 80.8% of negatives and 78.8% of positives.

The final RF model was evaluated on the independent test set. The receiver operating characteristic (ROC) curve yielded an AUC of 0.872 (**Figure 5B**). At the optimal probability cutoff of 0.48 (**Figure 5A**), the model achieved an accuracy of 0.795, sensitivity of 0.779, and specificity of 0.808. The cumulative gains curve demonstrated that the model captured approximately 80% of total fracture cases within the first 48.1% of the screened population (**Figure 5C**). The confusion matrix (**Figure 5D**) indicated balanced classification, correctly identifying 80.8% of non-fractured and 78.8% of fractured cases.

### 3.5 Determinants of fracture risk

**Figure 6:**
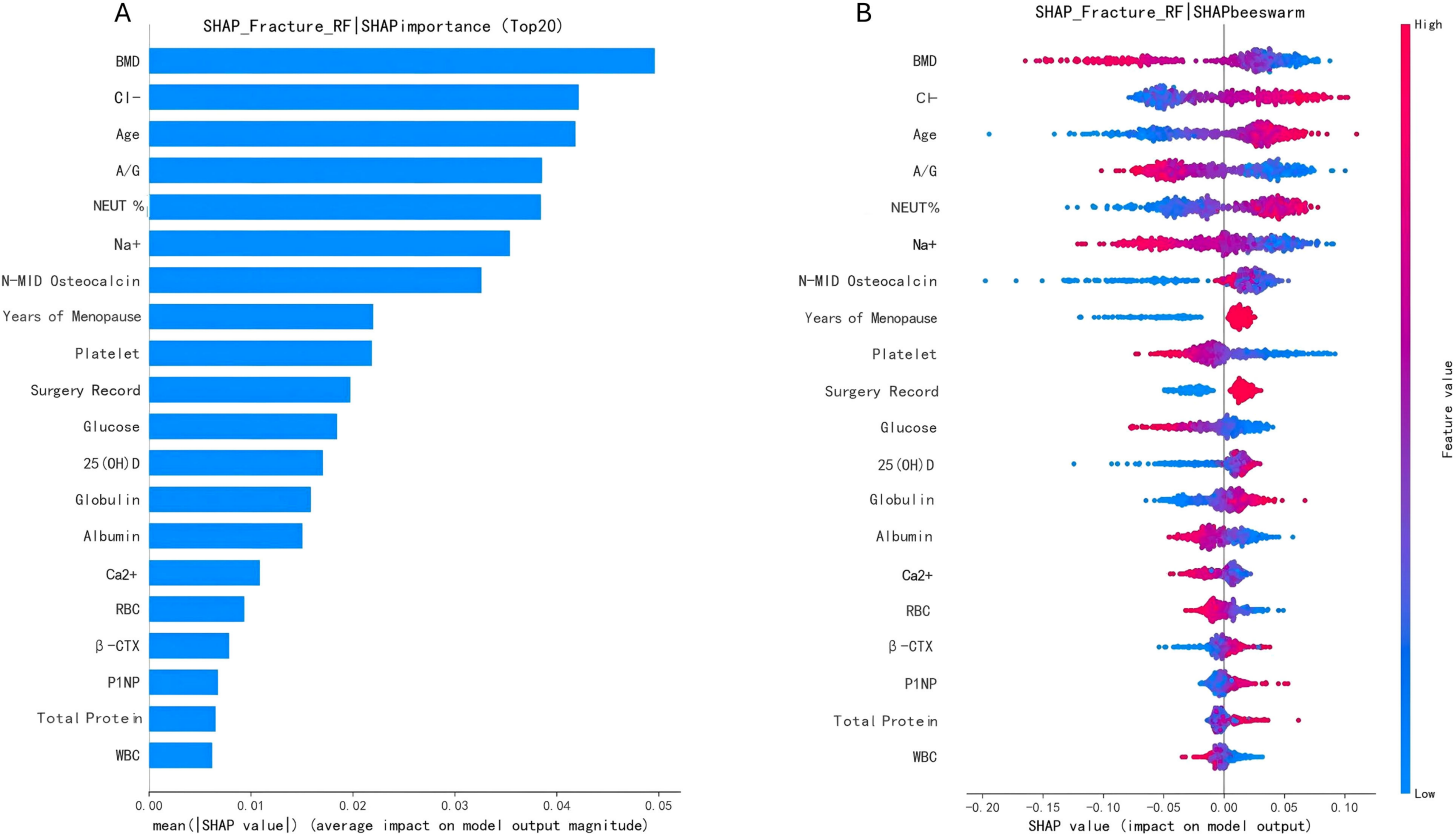
**(A)** Ranking of feature importance of the stacker model based on SHAP values. BMD: 0.0496; CI^-^:0.0422; Age: 0.0417; A/G: 0.0386; NEUT%: 0.0384; Na^+^: 0.0354; N-MID: 0.0326; Menopause years: 0.0220; Platelet: 0.0219; Sugar Record: 0.0198; FPG: 0.0185; 25(OH)D: 0.0171; GLB: 0.0159; ALB: 0.0151; Ca^2+^: 0.0109; RBC: 0.009; β-CTX: 0.008; P1NP: 0.007; TP: 0.007; WBC: 0.006. **(B)** Distribution of the impact of each feature on the output of the stacker model estimated using the SHAP values. The plot sorts the features by the sum of SHAP value magnitudes over all samples and shows the order of feature importance. This figure illustrates data from the test cohort, with each point representing a single patient. The color represents the feature value (red high, blue low). The x-axis measures the impact on the model output (right positive, left negative).

SHAP analysis of the RF model identified BMD as the most influential predictor of fracture risk. Serum chloride (Cl^-^) concentration emerged as the second most important feature, followed by age, albumin-to-globulin ratio, and neutrophil percentage (**Figure 6A**). Among bone turnover markers, N-MID demonstrated substantially greater contribution (ranked 7th overall) than β-CTX (17th) and P1NP (18th). The directional relationships between feature values and fracture risk are visualized in **Figure 6B**, where higher values of N-MID, β-CTX, and P1NP were associated with increased predicted fracture probability.

## 4 Discussion

Early identification of postmenopausal women at high risk of osteoporotic fractures is essential for timely intervention to prevent bone loss, reduce fracture incidence, and improve quality of life while potentially lowering healthcare costs (31). Conventional fracture risk assessment, largely dependent on BMD, has limited predictive accuracy and may miss a considerable proportion of at-risk individuals. In this study, we developed a RF model that integrates multidimensional clinical, biochemical, and imaging features, achieving an AUC of 0.872 and providing a more comprehensive tool for fracture risk stratification.

A key observation from our SHAP analysis is that N-MID contributed more to fracture risk prediction than the IOF/IFCC-recommended markers β-CTX and P1NP. This suggests that N-MID may capture aspects of bone turnover that are particularly relevant to bone quality and fracture susceptibility in postmenopausal women. N-MID is a fragment of osteocalcin involved in bone matrix mineralization and collagen cross-linking. Hence, its altered circulating levels may reflect disturbances in these processes, but the exact mechanisms remain to be elucidated in experimental studies (32–34). The comparatively weaker predictive value of β-CTX and P1NP in our cohort could be related to metabolic heterogeneity, including the influence of adipokines and low-grade inflammation on osteoclast activity (35). These findings underscore the value of including N-MID alongside traditional BTMs in future risk prediction models.

Serum Cl^-^ emerged as the second most important predictor after BMD. While the exact biological link between Cl- homeostasis and bone fragility remains unclear, several plausible hypotheses exist. Preclinical studies have suggested that the Cl- channel CLC-7/Ostm1 complex on osteoclast membranes is essential for acidification of the resorption lacuna, and that extracellular Cl^-^ concentrations may modulate this process (36). In addition, Cl- imbalance might impair osteocyte mechanosensing via connexin 43 (37). Our observation that elevated Cl^-^ (>107 mmol/L) substantially increased fracture risk, even in women with BMD near the osteoporotic threshold (0.70-- 0.75 g/cm²), raises the possibility of a synergistic effect. This finding should be considered hypothesis-generating and requires validation in independent cohorts and mechanistic studies.

Age and A/G ratio reflect cumulative physiological decline. Consistent with previous studies (38, 39), age was a strong predictor, with risk increasing progressively after 70 years. This trajectory likely represents the composite impact of age-related changes in bone metabolism, muscle mass, vitamin D synthesis, and inflammatory status (40, 41). The A/G ratio, an established marker of nutritional status and systemic inflammation, was also among the top predictors. Lower A/G ratio reflects hypoalbuminemia and relative hyperglobulinemia, which are associated with reduced bone formation and enhanced osteoclast activity (42). Recent cohort studies have similarly reported associations between low albumin or high globulin and increased fracture risk (43). The interaction between low A/G ratio and elevated N-MID observed in our study suggests a vicious cycle linking inflammation, malnutrition, and accelerated bone loss, but this remains a hypothesis to be tested prospectively.

Several ML-based fracture risk models have been reported, most of which rely predominantly on BMD, FRAX components, or imaging parameters (12, 42). Our study contributes unique novelty in three aspects. First, we systematically compared the predictive importance of three BTMs and demonstrated that N-MID outperforms β-CTX and P1NP, challenging the current IOF/IFCC emphasis on the latter two markers for risk prediction. Second, we identified serum Cl^-^ as a potentially modifiable biochemical predictor that has been largely overlooked in osteoporosis research. Third, we employed SHAP to provide transparent, individualized interpretations of model predictions, thereby enhancing clinical trustworthiness. To our knowledge, this is a novel study integrating N-MID, serum Cl^-^, and A/G ratio into a single ML framework for postmenopausal fracture prediction. Our model’s AUC (0.872) compares favorably with previously published models (e.g., AUCs ranging from 0.70 to 0.85) (42), although direct cross-study comparisons are hampered by differences in populations and predictor sets. Future work should prospectively compare our model with FRAX^®^ and assess its incremental clinical utility.

## 6 Limitations

Several limitations should be considered. First, as this study focused on biomarker exploration, the model was not directly compared with the clinical gold standard FRAX^®^, leaving its incremental value within existing frameworks to be clarified. Second, the cohort mainly comprised Han Chinese postmenopausal women from Northwestern China, limiting generalizability to other ethnic or regional populations. Third, the retrospective design relying on electronic medical records did not capture dynamic lifestyle factors such as falls, diet, or physical activity, potentially omitting relevant predictors. Finally, model validation was restricted to internal and local external datasets, lacking multicentric prospective confirmation. Future studies should perform multi-population prospective validations, incorporate dynamic variables, and evaluate integration with FRAX® in real-world clinical settings.

## 7 Conclusion

This study developed a machine learning–based model integrating multidimensional clinical features to predict osteoporotic fracture risk in postmenopausal women. N-MID contributed more to fracture prediction than the IOF/IFCC-recommended β-CTX and P1NP, highlighting its central role in reflecting dynamic bone turnover. Serum Cl^-^ was identified as the second strongest predictor after BMD, and its interaction with BMD allowed identification of high-risk individuals potentially missed by conventional assessment. Age and the A/G ratio further reflected the combined impact of aging and systemic inflammation on bone health. These findings emphasize the clinical value of integrating bone density, biochemical markers, electrolytes, and systemic indicators into a unified model for early identification and management of high-risk postmenopausal women.

## Data Availability

The raw data supporting the conclusions of this article will be made available by the authors, without undue reservation.

## References

[1] Long G Liu C Liang T Zhang Z Qin Z Zhan X . Predictors of osteoporotic fracture in postmenopausal women: a meta-analysis. J Orthop Surg Res. (2023) 18:574. doi: 10.1186/s13018-023-04051-6. PMID: 37543616 PMC10404374

[2] Qu X Wang Q Luo C Li Y Tian L Xu L . Association between bone turnover markers and FRAX predicted fracture risk in Chinese adults: a cross-sectional study. BMC Musculoskeletal Disord. (2025) 26:467. doi: 10.1186/s12891-025-08571-6. PMID: 40361069 PMC12070548

[3] Cooper C Ferrari S Reginster JY Dawson-Hughes B Rizzoli R Kanis JA . IOF compendium of osteoporosis. Nyon, Switzerland: International Osteoporosis Foundation (2017).

[4] Meng S Tong M Yu Y Cao Y Tang B Shi X . The prevalence of osteoporotic fractures in the elderly in China: a systematic review and meta-analysis. J Orthop Surg Res. (2023) 18:536. doi: 10.1186/s13018-023-04030-x. PMID: 37501170 PMC10373275

[5] Singer A McClung MR Tran O Morrow CD Goldstein S Kagan R . Treatment rates and healthcare costs of patients with fragility fracture by site of care: a real-world data analysis. Arch Osteoporos. (2023) 18:42. doi: 10.1007/s11657-023-01229-7. PMID: 36905559 PMC10008255

[6] Hernlund E Svedbom A Ivergård M Compston J Cooper C Stenmark J . Osteoporosis in the European Union: medical management, epidemiology and economic burden. Arch Osteoporos. (2013) 8:136. doi: 10.1007/s11657-013-0136-1. PMID: 24113837 PMC3880487

[7] Shen Y Huang X Wu J Lin X Zhou X Zhu Z . The global burden of osteoporosis, low bone mass, and its related fracture in 204 countries and territories, 1990-2019. Front Endocrinol. (2022) 13:882241. doi: 10.3389/fendo.2022.882241. PMID: 35669691 PMC9165055

[8] International Osteoporosis Foundation . Epidemiology Nyon: International Osteoporosis Foundation. (2025). Available from: https://www.osteoporosis.foundation/health-professionals/about-osteoporosis/epidemiology (Accessed February 4, 2026)

[9] Si L Winzenberg TM Jiang Q Chen M Palmer AJ . Projection of osteoporosis-related fractures and costs in China: 2010–2050. Osteoporos Int. (2015) 26:1929–37. doi: 10.1007/s00198-015-3093-2. PMID: 25761729

[10] Mousavibaygei MS Exuzides A Spangler L . Outdoor air pollution exposure, bone mineral density, osteoporosis, and osteoporotic fractures: a systematic review and meta-analysis. Sci Total Environ. (2022) 865:161117. doi: 10.1016/j.scitotenv.2022.161117. PMID: 36586679

[11] Song M Wang Y Jiang Y Pi H Lyu H Gao Y . Risk factors for subsequent fractures in hip fracture patients: a nested case-control study. J Orthop Surg Res. (2024) 19:348. doi: 10.1186/s13018-024-04833-6. PMID: 38867268 PMC11167847

[12] Tu JB Liao WJ Liu WC Gao XH . Using machine learning techniques to predict the risk of osteoporosis based on nationwide chronic disease data. Sci Rep. (2024) 14:5245. doi: 10.1038/s41598-024-56114-1. PMID: 38438569 PMC10912338

[13] Atiporn T Tanawat A . Trabecular bone score as a risk factor of major osteoporotic fracture in postmenopausal women: the first study in Thailand. J Menopausal Med. (2022) 283:112–20. doi: 10.6118/jmm.22011. PMID: 36647274 PMC9843030

[14] Huang W Cai XH Li YR Xu F Jiang XH Wang D . The association between paraspinal muscle degeneration and osteoporotic vertebral compression fracture severity in postmenopausal women. J Back Musculoskelet Rehabil. (2023) 36:323–9. doi: 10.3233/bmr-220059. PMID: 36155499 PMC10041424

[15] Luo W Zhang J Xu L Zhou Y Xu D Lv Q . Use of zoledronic acid in antiosteoporosis treatment is associated with a decreased blood lipid level in postmenopausal women with osteoporosis: a cohort study in China. Postgrad Med. (2022) 134:406–12. doi: 10.1080/00325481.2022.2051983. PMID: 35264059

[16] Chen CH Elsalmawy AH Ish-Shalom S Lim SJ AlAli NS Cunha-Borges JL . The effect of teriparatide treatment on the risk of fragility fractures in postmenopausal women with osteoporosis: results from the Asian and Latin America Fracture Observational Study (ALAFOS). Calcif Tissue Int. (2022) 110:74–86. doi: 10.1007/s00223-021-00895-4. PMID: 34415388 PMC8732800

[17] Palomo T Muszkat P Weiler FG Dreyer P Brandão CMA Silva BC . Update on trabecular bone score. Arch Endocrinol Metab. (2022) 66:694–706. doi: 10.20945/2359-3997000000559, PMID: 36382759 PMC10118821

[18] Okuwaki S Funayama T Ikumi A Matsuura S Kawamura H Yamazaki M . Relationship between vertebral instability and the cross-sectional area of lumbar muscles in postmenopausal acute osteoporotic vertebral fractures. Spine Surg Relat Res. (2022) 6:51–7. doi: 10.22603/ssrr.2021-0029. PMID: 35224247 PMC8842355

[19] Singh A Varma AR . Whole-body vibration therapy as a modality for treatment of senile and postmenopausal osteoporosis: a review article. Cureus. (2023) 15:e33690. doi: 10.7759/cureus.33690. PMID: 36793830 PMC9925023

[20] Murdoch TB Detsky AS . The inevitable application of big data to health care. JAMA. (2013) 309:1351–2. doi: 10.1001/jama.2013.393. PMID: 23549579

[21] Chen Y Hesse BW McBride D Agboola S Ilan E Ralston JD . Digital information ecosystems for modern care coordination and patient care pathways: enhancing collaboration and personalization with artificial intelligence. J Med Internet Res. (2024) 26:e60258. doi: 10.2196/60258, PMID: 39622048 PMC11650087

[22] Sung SF Lin CY Hu YH . EMR-based phenotyping of ischemic stroke using supervised machine learning and text mining techniques. IEEE J BioMed Health Inform. (2020) 24:2922–31. doi: 10.1109/jbhi.2020.2976931. PMID: 32142458

[23] Heo JN Yoon JG Park H Kim YD Nam HS Heo JH . Machine learning-based model for prediction of outcomes in acute stroke. Stroke. (2019) 50:1263–5. doi: 10.1161/strokeaha.118.024293. PMID: 30890116

[24] Castonguay AC Zoghi Z Zaidat OO Burgess RE Zaidi SF Mueller-Kronast N . Predicting functional outcome using 24-hour post-treatment characteristics: application of machine learning algorithms in the STRATIS registry. Ann Neurol. (2023) 93:40–9. doi: 10.1002/ana.26528. PMID: 36214566 PMC10091739

[25] Jo H Kim C Gwon D Lee J Lee J Park KM . Combining clinical and imaging data for predicting functional outcomes after acute ischemic stroke: an automated machine learning approach. Sci Rep. (2023) 13:16926. doi: 10.1038/s41598-023-44201-8. PMID: 37805568 PMC10560215

[26] World Health Organization (WHO) . Assessment of fracture risk and its application to screening for postmenopausal osteoporosis. Technical Report Series 843. Geneva: WHO (1994). 7941614

[27] Guan X Du Y Ma R Teng N Ou S Zhao H . Construction of the XGBoost model for early lung cancer prediction based on metabolic indices. BMC Med Inform Decis Mak. (2023) 23:107. doi: 10.1186/s12911-023-02171-x. PMID: 37312179 PMC10262551

[28] Ma J Bo Z Zhao Z Yang J Yang Y Li H . Machine learning to predict the response to Lenvatinib combined with Transarterial Chemoembolization for unresectable hepatocellular carcinoma. Cancers (Basel). (2023) 15:625. doi: 10.3390/cancers15030625. PMID: 36765583 PMC9913670

[29] Yang PT Wu WS Wu CC Shih YN Hsieh CH Hsu JL . Breast cancer recurrence prediction with ensemble methods and cost-sensitive learning. Open Med (Wars). (2021) 16:754–68. doi: 10.1515/med-2021-0282. PMID: 34027105 PMC8122465

[30] Bangalore Yogananda CG Shah BR Vejdani-Jahromi M Nalawade SS Murugesan GK Yu FF . A fully automated deep learning network for brain tumor segmentation. Tomography. (2020) 6:186–93. doi: 10.18383/j.tom.2019.00026. PMID: 32548295 PMC7289260

[31] Yu EW Tsourdi E Clarke BL Bauer DC Drake MT . Osteoporosis management in the era of COVID-19. J Bone Mineral Res. (2020) 35:1009–13. doi: 10.1002/jbmr.4049. PMID: 32406536 PMC7273005

[32] Guo X Shen Y Du T He Y Lu J Yang Q . Elevations of N-terminal mid-fragment of osteocalcin are associated with abnormal bone metabolism and decreased bone mineral density. J Physiol Investig. (2024) 67(6):335–343. doi: 10.4103/ejpi.EJPI-D-24-00042, PMID: 39311455

[33] Saito M Marumo K Nakaoka H Kato Y Kawai M Okada Y . Osteocalcin fragments as biomarkers of bone microstructure damage. Nat Commun. (2023) 14:1122. 36854674

[34] Jiang C Zhu S Zhan W Lou L Li A Cai J . Comparative analysis of bone turnover markers in bone marrow and peripheral blood: implications for osteoporosis. J Orthop Surg Res. (2024) 19:163. doi: 10.1186/s13018-024-04634-x, PMID: 38429649 PMC10908102

[35] Chen P Zhang Y Wang J Li X Liu Q Zhang H . Adiponectin inhibits osteoclastogenesis via AMPK-STAT3. Cell Metab. (2023) 36:1–15. doi: 10.1074/jbc.m110.152405. PMID: 21300805 PMC3069456

[36] Zhang S Liu Y Zhang B Zhou J Li T Liu Z Li Y . Molecular insights into the human CLC-7/Ostm1 transporter. Sci Adv. (2020) 6:eabb4747. doi: 10.1126/sciadv.abb4747, PMID: 32851177 PMC7423370

[37] Ma L . Connexin 43 in the function and homeostasis of osteocytes. Ann Joint. (2021) 9:10. doi: 10.21037/atm-22-6643. PMID: . 38529291 PMC10929443

[38] Ye C Morin SN Lix LM McCloskey EV Johansson H Harvey NC . Age at first fracture and later fracture risk in older adults undergoing osteoporosis assessment. JAMA Netw Open. (2024) 7:e2448208. doi: 10.1001/jamanetworkopen.2024.48208. PMID: 39621347 PMC11612869

[39] Langsetmo L Schousboe JT Taylor BC Cauley JA Fink HA Cawthon PM . Characteristics associated with 5-year fracture risk vs. 5-year mortality risk among late-life men. J Gerontol A Biol Sci Med Sci. (2022) 78:683–9. doi: 10.1093/gerona/glac159. PMID: 35917212 PMC10061558

[40] Yang Z Xu J Kang T Chen X Zhou C . The impact of NLRP3 inflammasome on osteoblasts and osteogenic differentiation: a literature review. J Inflammation Res. (2024) 17:2639–53. doi: 10.2147/jir.s457927. PMID: 38707958 PMC11067939

[41] Hao L Yan Y Huang G Li H . From gut to bone: deciphering the impact of gut microbiota on osteoporosis pathogenesis and management. Front Cell Infect Microbiol. (2024) 14:1416739. doi: 10.3389/fcimb.2024.1416739. PMID: 39386168 PMC11461468

[42] Kim Y Kim YG Park JW Kim BW Shin Y Kong SH . A CT-based deep learning model for predicting subsequent fracture risk in patients with hip fracture. Radiology. (2024) 310:e230614. doi: 10.1148/radiol.230614. PMID: 38289213

[43] Li Q Zhu M Liu X Tian C Li D Wang H . Abnormally low serum albumin levels are associated with abnormal bone mineral density and osteoporotic fractures: a retrospective studies. BMC Musculoskelet Disord. (2024) 25:888. doi: 10.1186/s12891-024-08021-9. PMID: 39511536 PMC11542386

